# Supplementary material for: The cell envelope of Staphylococcus aureus selectively controls the sorting of virulence factors
Source: Nat Commun. 2021 Oct 26;12:6193. doi: 10.1038/s41467-021-26517-z (PMC8548510; doi:10.1038/s41467-021-26517-z)
Supplement: Supplementary file 1 — Supplementary Information [file 41467_2021_26517_MOESM1_ESM.pdf]

**Supplementary Information for the manuscript: The cell envelope of *Staphylococcus aureus* selectively controls the sorting of virulence factors**

## Figures and Figure Legends

```
              Signal sequence            Cleavage site          Mature protein

>LukA MKNKKRV----LIASSLSCAILLLSAATTQANS--|AHKDSQDQNKKEHVDKSQQKDKRNVTNKDKNSTAPD
>LukS MVKKRLL------AATLSLGIITPIATSFHESKA-|-------------------------------DNNIE
>LukE MFKKKML------AATLSVGLIAPLASPIQESRA-|-------------------------------NTNIE
>HlgA MIKNKIL------TATLAVGLIAPLANPFIEISKA|-------------------------------ENKIE
>HlgC MLKNKIL------TTTLSVSLLAPLANPLLENAKA|-------------------------------ANDTE
>LukB -MIKQLCKNITICTLALSTTFTVLPATSFA-----|--------------------------KINSEIKQVS
>LukF --MKKIV----KSSVVTSIALLLLSNTVDA-----|----------------------------AQHITPVS
>LukD MKMKKLV----KSSVASSIALLLLSNTVDA-----|----------------------------AQHITPVS
>HlgB MKMNKLV----KSSVATSMALLLLSGTANA-----|----------------------------EGKITPVS
```

**Supplementary Figure 1. Related to Figure 1. Prediction of signal sequence in leukocidins.**
SignalP 5.0 prediction of the Sec signal sequence in all leukocidin subunits. Potential cleavage sites (probability > 0.1) are shown in bold. The signal sequences and the mature proteins were aligned separately using Clustal Omega with default settings. Only the N-terminus of mature protein alignment is shown.

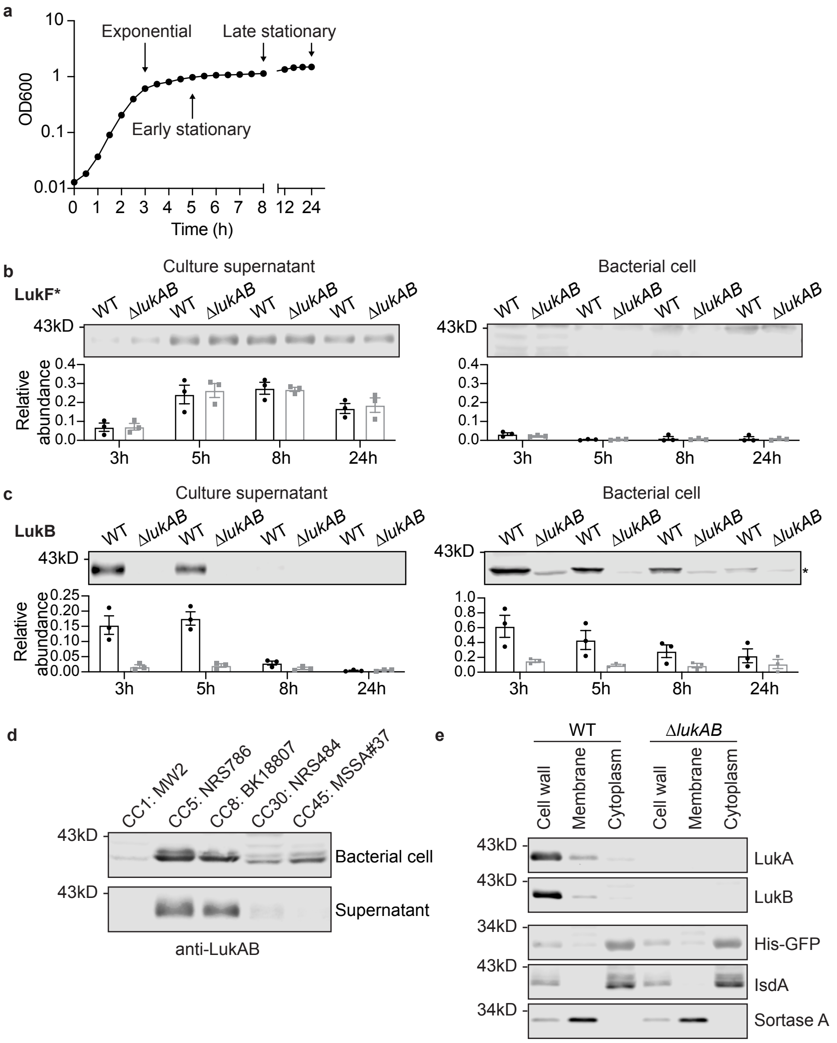


**Supplementary Figure 2. Related to Figure 1. The cell association of LukAB is consistent in the clinical isolates.**

(a) Growth curve of *S. aureus* in TSB. Data show mean ± SEM from 8 independent experiments. Arrows indicate the time points used in this work.

(b) Detection of the F-subunit of other leukocidins in the culture supernatant and the bacterial cell lysate at different growth phases. Data show a representative immunoblot (top) and mean ± SEM of the protein signal (bottom) from 3 independent experiments. Targeted protein signals were identified and normalized to 50 ng of purified LukD. LukF*, the antibody recognizes LukF-PV, LukD, and HlgB. These subunits are highly similar in length and protein sequence. The representative immunoblots were adjusted to the same brightness/contrast.

(c) Detection of LukB in the culture supernatant and the bacterial cell lysate at different growth phases. Data show a representative immunoblot (top) and mean ± SEM of the protein signal (bottom) from 3 independent experiments. Targeted protein signals were identified and normalized to 50 ng of purified LukAB. *, non-specific binding of the anti-LukB antibody.

(d) Immunoblot of LukAB in the bacterial cell lysate and the culture supernatant of a spectrum of clinical isolates with a mixture of monoclonal antibodies which can recognize LukAB across different *S. aureus* lineages. Representative immunoblots of 2 independent experiments are shown.
(e) Immunoblot of LukA, LukB, His-GFP, IsdA, and Sortase A in the cell wall, membrane, and cytoplasm fractions of USA300. Representative immunoblots of 2 independent experiments are shown.
Source data are provided as a Source Data file.

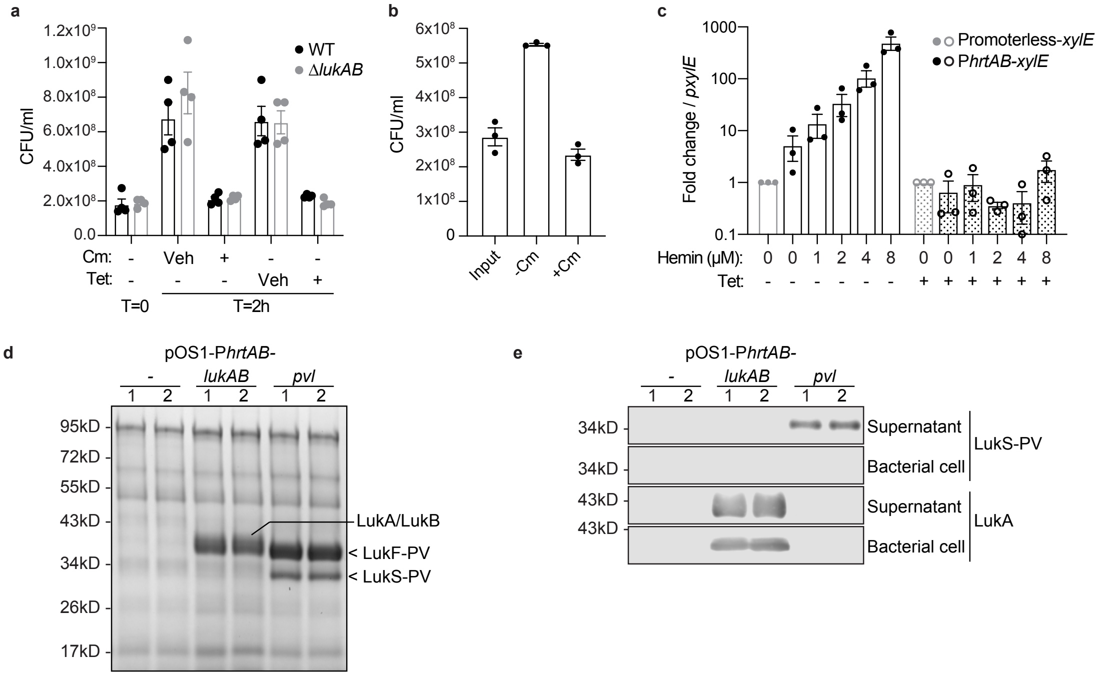


**Supplementary Figure 3. Related to Figure 2. Chloramphenicol and tetracycline inhibit USA300 growth and protein translation*.***

(a) CFUs of USA300 after incubation ± 100 μg/ml chloramphenicol (Cm) or 40 μg/ml tetracycline (Tet) for 2 h. Veh, vehicle control for each antibiotic. Bars indicate mean ± SEM of 5 independent experiments.

(b) CFUs of USA300 after incubation ± 100 μg/ml Cm in the presence of PMNs for 2 h. Bars indicate mean ± SEM of 3 independent experiments.

(c) Promoter activity of *hrtAB*. WT USA300 expressing *xylE* under P*hrtAB* or no promoter control was incubated with different concentrations of hemin ± 40 μg/ml tetracycline (Tet). XylE activity was measured after 1h. Bars indicate mean ± SEM of 3 independent experiments.

(d) Coomassie stained SDS-PAGE of the secreted proteins in a leukocidin-null isogenic mutant strain complemented with hemin-inducible *lukAB* or *pvl*. The MW of target proteins: LukA, 37 kDa; LukB, 36 kDa; LukF-PV, 34 kDa; LukS-PV, 32 kDa.

(e) Immunoblot of LukS-PV and LukA in the culture supernatant and the bacterial cell lysate of a leukocidin-null isogenic mutant strain complemented with hemin-inducible *lukAB* or *pvl*. The immunoblots were adjusted to the same brightness/contrast for each target.

(d-e) The exoproteins and leukocidins profiles were examined with 2 independent colonies in one gel or immunoblot.

Source data are provided as a Source Data file.

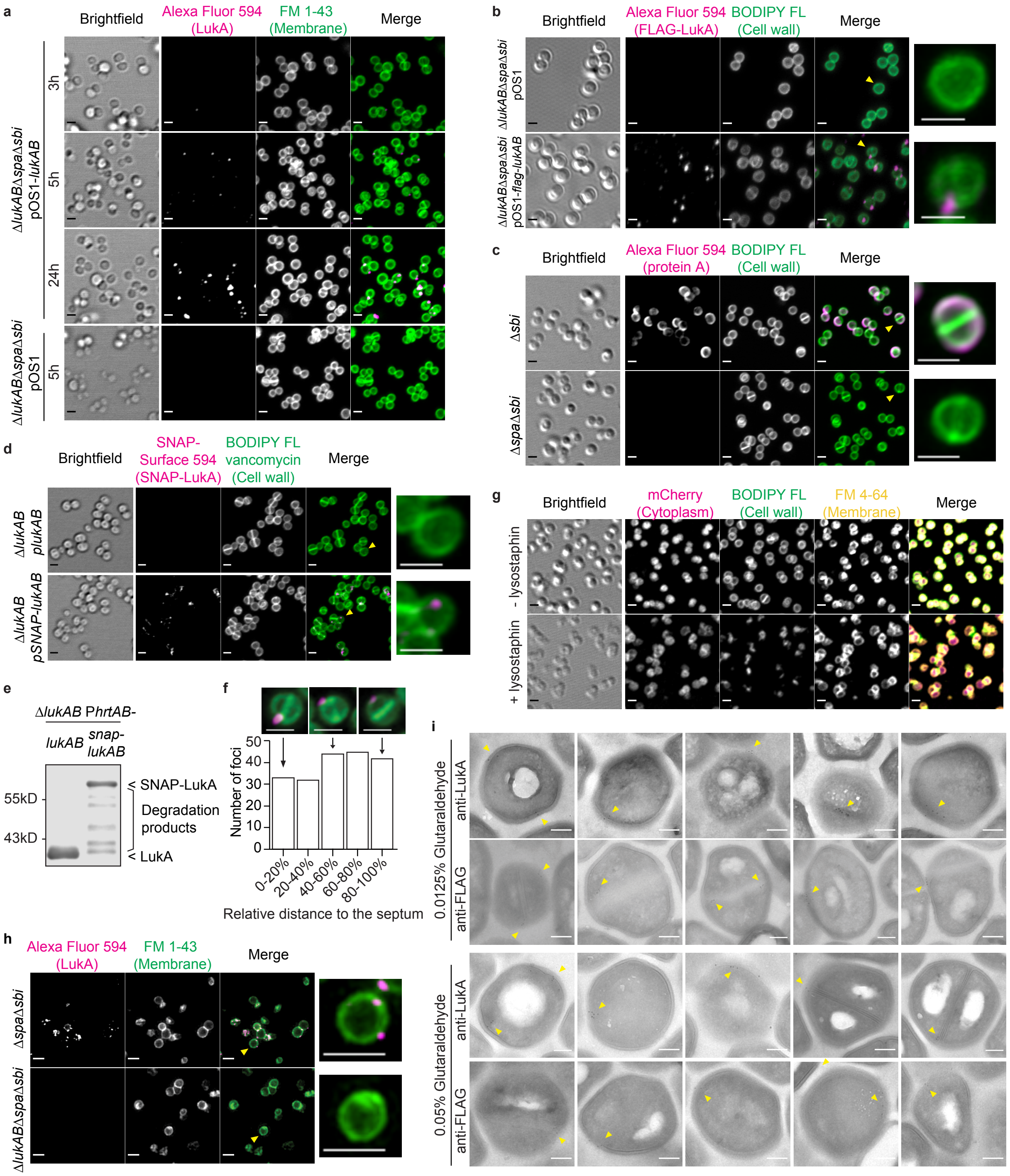


**Supplementary Figure 4. Related to Figure 3. LukAB clusters as discrete foci on USA300 cells.**

(a) Immunofluorescent imaging of LukA on USA300 at the exponential phase (3h), early stationary phase (5h), and late stationary phase (24h). The pOS1-*lukAB* plasmid over-expressed *lukAB* and pOS1 is the empty vector. The cell wall was stained with BODIPY FL-vancomycin. Representative images of 3 independent experiments are shown.

(b) Immunofluorescent imaging of FLAG-tagged LukA on USA300 at the late stationary phase (24 h). Δ*lukAB*Δ*spa*Δ*sbi* was complemented by expressing 3xFLAG-tagged *lukAB* in a plasmid (pOS1-*flag-lukAB*). The cell wall was stained with BODIPY FL vancomycin. One replicate of this experiment was performed.
(c) Immunofluorescent imaging of protein A on USA300 at the late stationary phase (24 h). A Δ*sbi* strain was used to minimize unspecific staining, and the Δ*spa*Δ*sbi* is the negative control. The cell wall was stained with BODIPY FL vancomycin. Representative images of 2 independent experiments are shown.
(d) Imaging of SNAP-tagged LukAB on the surface at the early stationary phase (5h). The SNAP-tagged LukAB was induced by 2 μM hemin and stained with SNAP-surface 594. The cell wall was stained with BODIPY FL vancomycin. Representative images of 2 independent experiments are shown.
(e) Immunoblot of native LukA and SNAP-tagged LukA on the USA300 surface. The samples were prepared from the same cells imaged in (d). A representative immunoblot of 2 independent experiments is shown.
(f) Histogram of the number of foci at different distances to the closest septum. Example pictures above show 0%, 50%, and 100% distance. A total of 196 foci found in cells with clear septum were pooled from 6 independent experiments and analyzed.
(g) Imaging of USA300 cells treated with lysostaphin. A USA300 strain expressing mCherry in the cytoplasm was treated ± lysostaphin, and then stained with BODIPY FL vancomycin for the cell wall and FM 4-64 for the membrane. Representative images of 2 independent experiments are shown.
(h) N-SIM images of LukA immunostaining on lysostaphin treated bacterial cells at the early stationary phase (5 h). Representative images of 2 independent experiments are shown.
(a-d, f-h) For fluorescent channels (FM 1-43, BODIPY FL, Alexa Fluor 594, and SNAP-surface 594), the maximum projection of Z-stack images is shown. The brightfield image is a single Z slice. Yellow arrows point to the single cells shown in enhanced images on the right. Scale bar, 1 μm.
(i) TEM images showing the immunogold labeling of LukAB. The section was probed with antibodies against FLAG-tag or LukA. Two different concentrations of glutaraldehyde were used for fixation. Yellow arrows indicate clusters of gold particles. Scale bar, 200 nm. Representative images of 2 independent EM sessions with different glutaraldehyde concentrations are shown.
Source data are provided as a Source Data file.

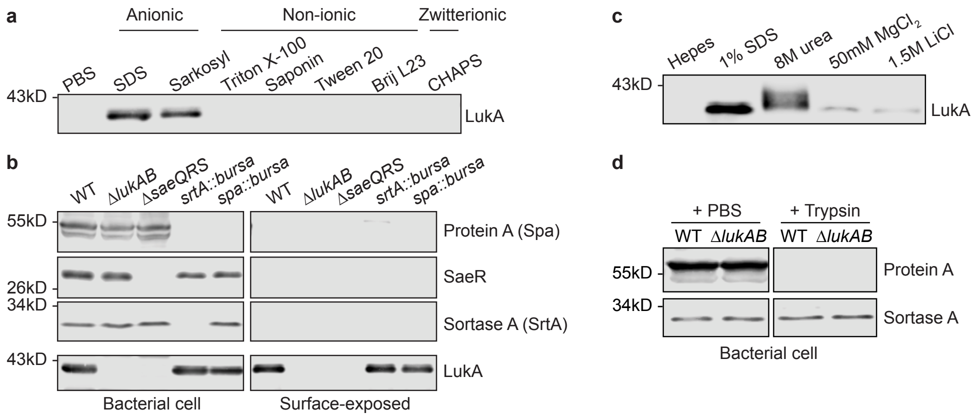


**Supplementary Figure 5. Related to Figure 4. SDS and trypsin extract proteins exposed on the surface.**

(a) Immunoblot of LukA in the cell-free supernatant after solubilization with different detergents in PBS. A representative immunoblot of 2 independent experiments is shown.

(b) Immunoblot of protein A (cell wall), SaeR (cytoplasm), sortase A (membrane), and LukA in the bacterial cell lysate and the SDS-released fraction in the bacterial cell (the surface-exposed compartment). One replicate of this experiment was performed.

(c) Immunoblot of LukA in the cell-free supernatant after solubilization with indicated reagents in 25 mM Hepes. A representative immunoblot of 2 independent experiments is shown.

(d) Immunoblot of protein A and sortase A in the bacterial cell lysate ± trypsin pre-treatment. One replicate of this experiment was performed.

Source data are provided as a Source Data file.

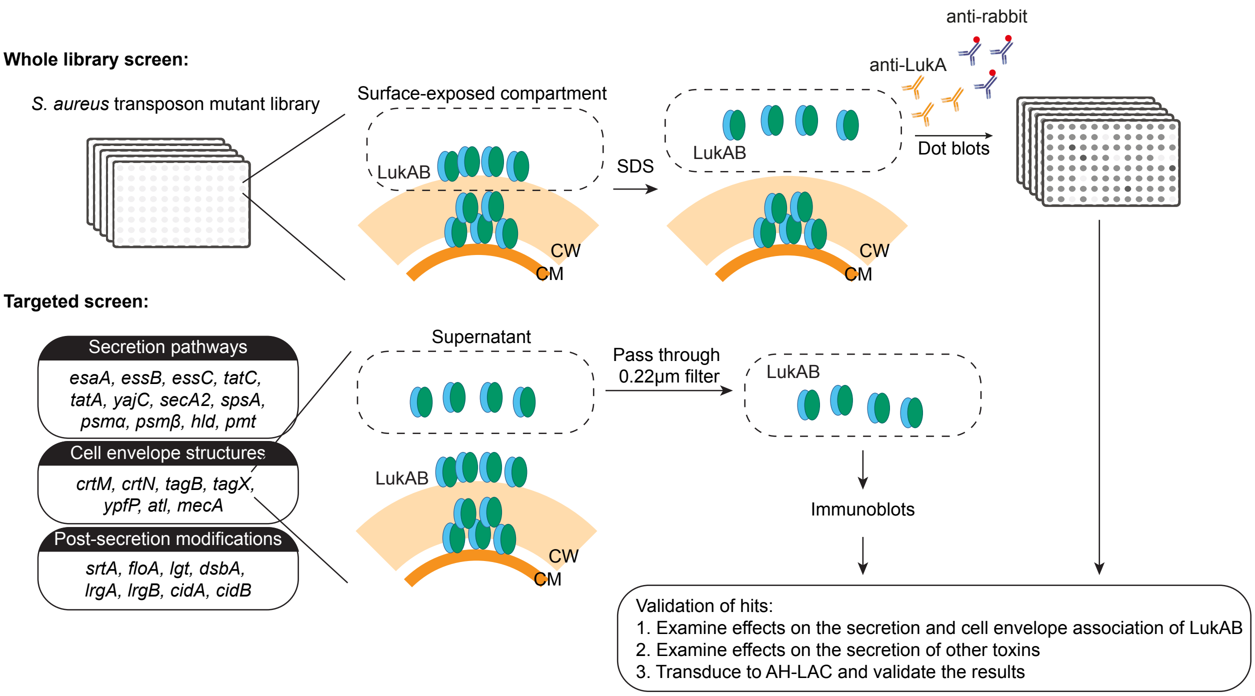


**Supplementary Figure 6. Related to Figure 5. Diagram of genetic screens to find factors that influence LukAB secretion.**

In the whole library screen, proteins in the surface-exposed compartment of the entire library were solubilized with SDS and LukA levels were measured by dot blots. In the targeted screen, proteins in the culture supernatant of selected mutants were collected and LukA levels were measured by immunoblots. For validation, levels of LukAB and other toxins were examined by immunoblots. CW, cell wall and CM, cell membrane.

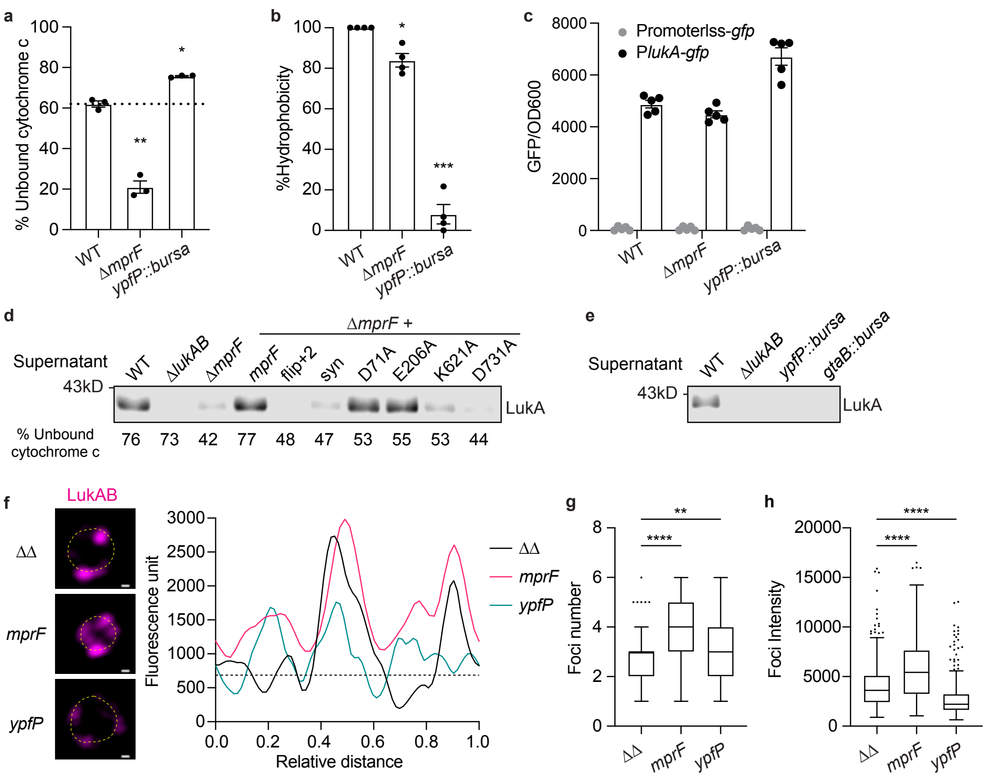


**Supplementary Figure 7. Related to Figure 5. Characterization of the *mprF* and *ypfP* mutants.**

(a) Surface charge of WT, *mprF* mutant, and *ypfP* mutant measured by cytochrome c binding. Higher unbound cytochrome c indicates that the surface is more positively charged. Bars indicate mean ± SEM from 3 independent experiments. **$p$=0.0064 and *$p$=0.022 compared to WT by RM one-way ANOVA with Dunnett's multiple comparison test.

(b) Surface hydrophobicity of WT, *mprF* mutant, and *ypfP* mutant. Data were normalized to WT. Bars indicate mean ± SEM from 4 independent experiments. *$p$=0.028 and ***$p$=0.0005 compared to WT by RM one-way ANOVA with Dunnett's multiple comparison test.

(c) Promoter activity of *lukAB* measured by *gfp* fused to the *lukAB* promoter. The GFP signal was normalized by OD600. Bars indicate mean ± SEM from 5 independent experiments.

(d) Immunoblot of LukA in the culture supernatant of the Δ*mprF* mutant complemented with flippase + 2 transmembrane domains, synthase domain, or full-length *mprF* with point mutations. The numbers at the bottom indicate the levels of positive charge on the surface of each strain, measured by cytochrome c binding. A representative immunoblot of 2 independent experiments is shown.

(e) Immunoblot of LukA in the culture supernatant of the *ypfP* and *gtaB* mutants. A representative immunoblot of 2 independent experiments is shown.

(f) Example single cell images of LukA immunofluorescence in the *mprF* and *ypfP* mutants in the background of USA300 strain lacking *spa* and *sbi* (ΔΔ, Δ*spa*Δ*sbi*, n=145; *mprF*, Δ*spa*Δ*sbi*Δ*mprF,* n=87; *ypfP*, Δ*spa*Δ*sbi/ypfP::spec,* n=97). Dashed line depicts the cell shape. Scale bar, 200 nm. The fluorescent intensity profiles of LukA were measured along the cell wall and shown on the right.

(g) Distribution of the number of LukAB foci per cell. Isolated and intact single cells from 3 independent experiments were analyzed (ΔΔ, Δ*spa*Δ*sbi*, n=145; *mprF*, Δ*spa*Δ*sbi*Δ*mprF,* n=87; *ypfP*, Δ*spa*Δ*sbi/ypfP::spec,* n=97).

(h) Distribution of the intensity of each LukAB focus. Foci were detected on isolated and intact single cells from 3 independent experiments (ΔΔ, Δ*spa*Δ*sbi*, n=392; *mprF*, Δ*spa*Δ*sbi*Δ*mprF,* n=321; *ypfP*, Δ*spa*Δ*sbi/ypfP::spec,* n=311).

(g, h) Data are represented as boxplots where the box extends from the first and third quartiles, the middle line is the median, the whiskers extends to the smallest or largest value with no further than 1.5 times IQR (inter-quartile distance) from the hinges of the box, and data beyond the whiskers are plotted individually. **$p$=0.0014 and ****$p$<0.0001 compared to WT determined by ordinary one-way ANOVA with Dunnett's multiple comparison test.

Source data are provided as a Source Data file.

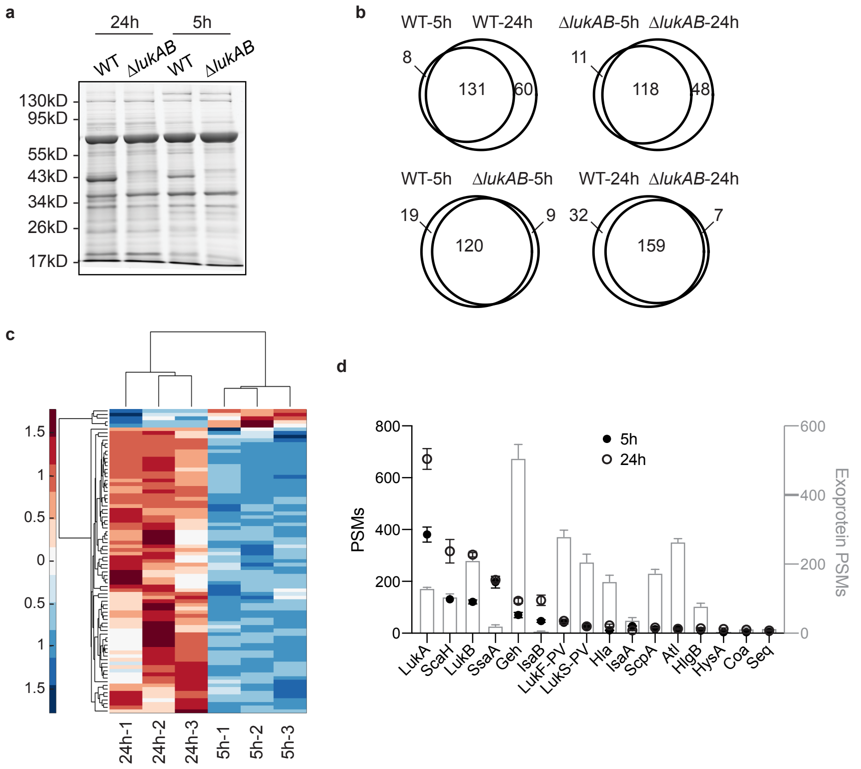


**Supplementary Figure 8. Related to Figure 6. The non-covalent association of proteins to the USA300 surface is influenced by growth phases.**

(a) SYPRO-Ruby stained SDS-PAGE of representative samples used in the proteomics study. The bands close to 43 kDa that are missing in the Δ*lukAB* correspond to LukA and LukB. A representative image of 3 independent samples preparations is shown.

(b) Venn diagrams of proteins identified in each condition.

(c) Heatmap of protein abundance in WT samples with unsupervised hierarchical clustering. Proteins whose peptide spectrum matches (PSMs) are statistically different between the early stationary phase (5h) and the late stationary phase (24h) are shown ($p \leq 0.05$ determined by two-tailed *t* tests). Each row is a different protein and each column is an individual sample. Color scales indicate the z-scores of protein levels across samples. (Raw data are provided in Supplementary Data 2)

(d) Predicted extracellular proteins on the surface of USA300. Circles (left axis) represent the PSMs of proteins identified on the bacterial surface in our proteomics study. Bars (right axis) indicate the PSMs in the culture supernatant from a previous study [28]. The two studies used the same bacterial strain and growth condition. The figure depicts that the relative composition of the two proteomes differs and is not correlated. Data show mean ± SEM of 3 independent samples.

Source data are provided as a Source Data file.

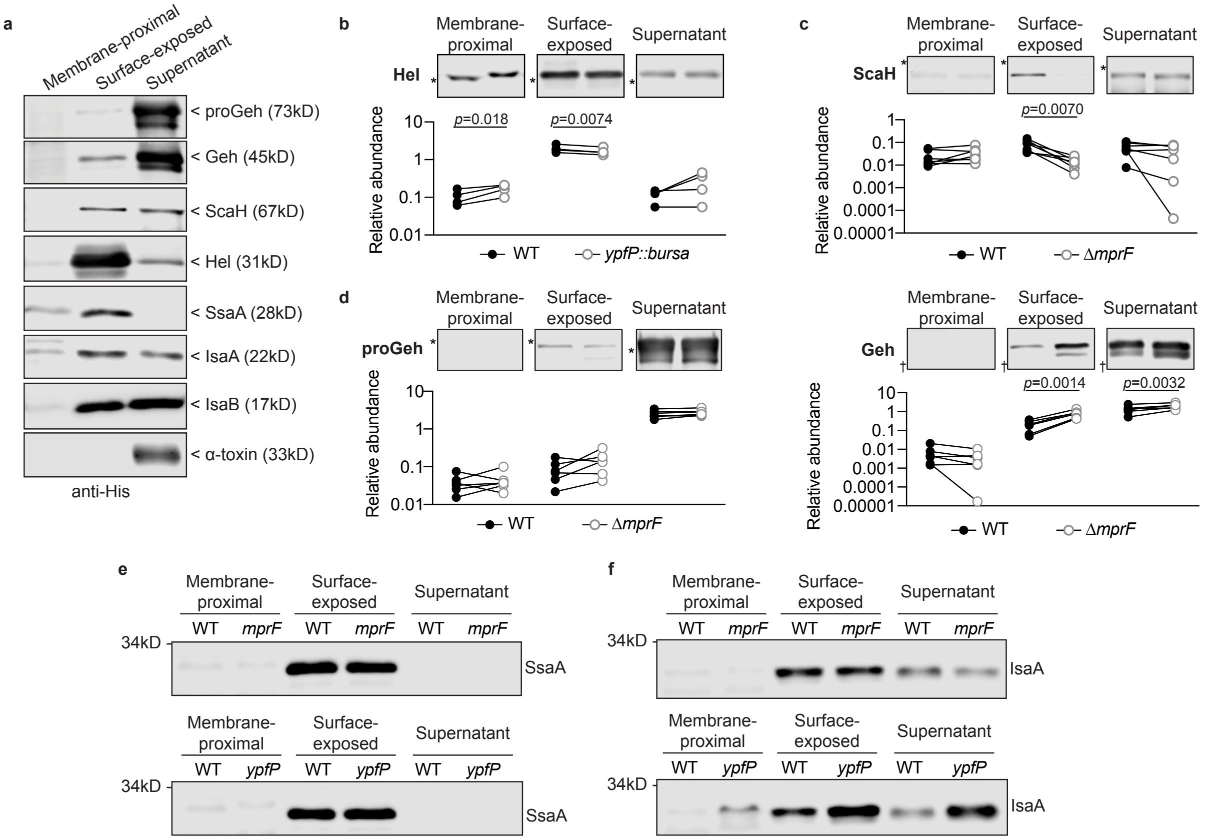


**Supplementary Figure 9. Related to Figure 6. Effects of MprF and YpfP-LtaA on protein localization.**

(a) Immunoblot of His-tagged Geh, ScaH, Hel, SsaA, IsaA, IsaB, and α-toxin in the membrane-proximal compartment, surface-exposed compartment, and culture supernatant at the early stationary phase.

(b) Representative immunoblot (top) and quantification (bottom) of His-tagged Hel in indicated fractions in WT and the *ypfP* mutant from 4 independent experiments. *, protein marker of 34 kD.

(c) Representative immunoblot (top) and quantification (bottom) of His-tagged ScaH in indicated fractions in WT and the *mprF* mutant from 7 independent experiments. *, protein marker of 95 kD.

(d) Representative immunoblot (top) and quantification (bottom) of His-tagged proGeh and Geh in indicated fractions in WT and the *mprF* mutant from 6 independent experiments. *, protein marker of 95 kD. †, protein marker of 43 kD.

(b-d) Each dot is an independent experiment. The protein signals were normalized against 50 ng purified His-tagged LukAB in each experiment. The *p*-values were determined by two-tailed paired *t* tests.

(e, f) Immunoblot of His-tagged SsaA (e) and IsaA (f) in WT, the *mprF*, and the *ypfP* mutants. Representative images of 2 independent experiments are shown.
