# Supplementary material for: The cell envelope of Staphylococcus aureus selectively controls the sorting of virulence factors
Source: Nat Commun. 2021 Oct 26;12:6193. doi: 10.1038/s41467-021-26517-z (PMC8548510; doi:10.1038/s41467-021-26517-z)
Supplement: Supplementary file 3 — Description of Additional Supplementary Files [file 41467_2021_26517_MOESM3_ESM.pdf]

Description of Additional Supplementary Files

File Name: Supplementary Data 1
Description: Screen data.

File Name: Supplementary Data 2
Description: Surface proteomics.

File Name: Supplementary Data 3
Description: Bacterial strains, plasmids, and primers.
