# Supplementary material for: The cell envelope of Staphylococcus aureus selectively controls the sorting of virulence factors
Source: Nat Commun. 2021 Oct 26;12:6193. doi: 10.1038/s41467-021-26517-z (PMC8548510; doi:10.1038/s41467-021-26517-z)
Supplement: Supplementary file 7 — Reporting Summary [file 41467_2021_26517_MOESM7_ESM.pdf]

nature research

Corresponding author(s): Victor J. Torres

Last updated by author(s): Sep 22, 2021

Reporting Summary

Nature Research wishes to improve the reproducibility of the work that we publish. This form provides structure for consistency and transparency in reporting. For further information on Nature Research policies, see our Editorial Policies and the Editorial Policy Checklist.

Statistics

For all statistical analyses, confirm that the following items are present in the figure legend, table legend, main text, or Methods section.

n/a	Confirmed	
☐	☒	The exact sample size (*n*) for each experimental group/condition, given as a discrete number and unit of measurement
☐	☒	A statement on whether measurements were taken from distinct samples or whether the same sample was measured repeatedly
☐	☒	The statistical test(s) used AND whether they are one- or two-sided<br>*Only common tests should be described solely by name; describe more complex techniques in the Methods section.*
☒	☐	A description of all covariates tested
☐	☒	A description of any assumptions or corrections, such as tests of normality and adjustment for multiple comparisons
☐	☒	A full description of the statistical parameters including central tendency (e.g. means) or other basic estimates (e.g. regression coefficient) AND variation (e.g. standard deviation) or associated estimates of uncertainty (e.g. confidence intervals)
☐	☒	For null hypothesis testing, the test statistic (e.g. *F*, *t*, *r*) with confidence intervals, effect sizes, degrees of freedom and *P* value noted<br>*Give P values as exact values whenever suitable.*
☒	☐	For Bayesian analysis, information on the choice of priors and Markov chain Monte Carlo settings
☒	☐	For hierarchical and complex designs, identification of the appropriate level for tests and full reporting of outcomes
☒	☐	Estimates of effect sizes (e.g. Cohen's *d*, Pearson's *r*), indicating how they were calculated

Our web collection on statistics for biologists contains articles on many of the points above.

Software and code

Policy information about availability of computer code

Data collection	Growth curve: The growth curve was measured using the Bioscreen C (Oy Growth Curves Ab Ltd).<br>Immunoblots: Immunoblots were imaged using the Odyssey Clx imaging system (Li-Cor Biosciences).<br>Intoxication/Infection/XylE reporter/GFP reporter/Cytochrome c binding/Hydrophobicity assays: The fluorescence or absorbance signals were measured using a 2103 Envision multilabel plate reader (PerkinElmer).<br>Microscopy: The images were obtained using a Zeiss 880 Laser Scanning Confocal microscope and the Zen software (Zeiss). The SIM images were obtained using a N-SIM S Super Resolution Microscope and the NIS-elements AR software (Nikon). The EM images were obtained using a Leo 912AB or Leo 906 transmission electron microscope (Zeiss) with a Cantega or Morada camera (SIS) and the ImageSP software from TRS (Tröndle).<br>Mass spectrometry: The MS/MS spectra were obtained using a Orbitrap Fusion Lumos mass spectrometer (ThermoFisher). The peptide identification was performed with the MaxQuant software suite (version 1.5.2.8).
Data analysis	GraphPad Prism 8 was used to plot data and carry out statistical analysis. Image studio (version 5.2.5, Li-Cor Biosciences) was used to analyze immunoblots and quantify protein levels. ImageJ (Fiji distribution) was used to analyze microscopy images. ImageJ plugin MicrobeJ (version 5.13l) was used to quantify the LukA foci. The mass spectrometry results were further analyzed with MATLAB and Venn function is acquired from MATLAB file exchange (https://www.mathworks.com/matlabcentral/fileexchange/22282-venn). SignalP-5.0 was used to predict signal sequences of leukocidins. Clustal Omega (EMBL-EBI) was used to align the signal sequences and mature proteins of leukocidins. PSORTb (version 3.0.2) was used to predict protein localizations. The ScanProsite (Expasy) and HMMER web server (version 2.41.1) were used to search for motifs present in LukAB.

For manuscripts utilizing custom algorithms or software that are central to the research but not yet described in published literature, software must be made available to editors and reviewers. We strongly encourage code deposition in a community repository (e.g. GitHub). See the Nature Research guidelines for submitting code & software for further information.

Data

Policy information about availability of data

All manuscripts must include a data availability statement. This statement should provide the following information, where applicable:

- Accession codes, unique identifiers, or web links for publicly available datasets
- A list of figures that have associated raw data
- A description of any restrictions on data availability

The mass spectrometric raw data generated in this study are accessible in the MassIVE database under ID: MSV000086238 (doi:10.25345/C5NF5S). The exoprotein mass spectrometry data were obtained from the MassIVE database under ID: MSV000080260. The nucleotide sequences of genes used this manuscript were acquired from NCBI SAUSA300_FPR3757 genome (NC_007793.1). Source data are provided with this paper. All other data supporting the key findings of this study are available from the corresponding author upon reasonable request.

Field-specific reporting

Please select the one below that is the best fit for your research. If you are not sure, read the appropriate sections before making your selection.

☒ Life sciences ☐ Behavioural & social sciences ☐ Ecological, evolutionary & environmental sciences

For a reference copy of the document with all sections, see nature.com/documents/nr-reporting-summary-flat.pdf

Life sciences study design

All studies must disclose on these points even when the disclosure is negative.

Sample size	No statistical methods were used to determine samples size. The sample size used are standards within the field and described within the methods or figure legends.
Data exclusions	No data points were excluded from analysis.
Replication	Multiple independent experiments were carried out and all attempts to reproduce data were successful. The number of independent experiments are listed in the figure legends.
Randomization	Allocation was random.
Blinding	Blinding was not relevant in this study, since all main conclusions were made based on quantitative results.

Reporting for specific materials, systems and methods

We require information from authors about some types of materials, experimental systems and methods used in many studies. Here, indicate whether each material, system or method listed is relevant to your study. If you are not sure if a list item applies to your research, read the appropriate section before selecting a response.

Materials & experimental systems

n/a	Involved in the study	
☐	☒	Antibodies
☒	☐	Eukaryotic cell lines
☒	☐	Palaeontology and archaeology
☐	☒	Animals and other organisms
☒	☐	Human research participants
☒	☐	Clinical data
☒	☐	Dual use research of concern

Methods

n/a	Involved in the study	
☒	☐	ChIP-seq
☒	☐	Flow cytometry
☒	☐	MRI-based neuroimaging

Antibodies

Antibodies used

1. Rabbit anti-LukA (generated in this lab, 1:5000)
2. Rabbit anti-LukB (generated in this lab, 1:1000)
3. Rabbit anti-LukS (generated in this lab, 1:5000)
4. Rabbit anti-LukF (generated in this lab, 1:5000)
5. Rabbit anti-Hla (Sigma-Aldrich, S7531, 1:5000)
6. Rabbit anti-IsdA (Gift of Dr. Eric Skaar, 1:25000)
7. Rabbit anti-sortase A (Gift of Dr. Eric Skaar, 1:20000)
8. Rabbit anti-SaeR (Gift of Dr. Taeok Bae, 1:2000)
9. Mouse anti-His (Cell Sciences, CSI20563C, 1:1000)

10. Affinity-purified rabbit anti-LukA (generated in this lab, 2.5µg/ml for intoxication assays, 10µg/ml for immunofluorescence, 5µg/ml for EM)
11. Goat anti-Rabbit, Alexa Fluor 680 (Invitrogen, A21076, 1:25000)
12. Goat anti-Mouse, Alexa Fluor 680 (Invitrogen, A21057, 1:25000)
13. Mouse anti-LukAB (CC8-1-4.3.1.2.5.3, CC30-3-10.1.5.9, or CC45-1-11.3.5, generated in this lab, 1µg/ml each)
14. Mouse anti-FLAG (Sigma, F1804, 1:100 for immunofluorescence, 1:200 for EM)
15. Rabbit anti-protein A (Sigma, P3775, 1:1000)
16. Goat Anti-Rabbit IgG H&L (Alexa Fluor 594) (Abcam, ab150080, 1:1000)
17. Goat Anti-Rabbit IgG H&L (Alexa Fluor 594) (Abcam, ab150116, 1:1000)
18. 6 nm Colloidal Gold AffiniPure Goat Anti-Mouse IgG (H+L) (Jackson ImmunoResearch, 115-195-146, 1:80)
19. 12 nm Colloidal Gold AffiniPure Goat Anti-Mouse IgG (H+L) (Jackson ImmunoResearch, 115-205-146, 1:80)
20. 12 nm Colloidal Gold AffiniPure Goat Anti-Rabbit IgG (H+L) (Jackson ImmunoResearch, 111-205-144, 1:80)

Validation

Antibodies were validated by the manufacturer, previous publication, or in this study by comparing specific signals to mutant strains:
1. Rabbit anti-LukA: 10.1111/j.1365-2958.2010.07490.x
2. Rabbit anti-LukB: 10.1111/j.1365-2958.2010.07490.x
3. Rabbit anti-LukS: 10.1111/j.1365-2958.2011.07942.x
4. Rabbit anti-LukF: 10.1128/mBio.02272-17
5. Rabbit anti-Hla: by manufacturer and in house studies such as 10.1111/j.1365-2958.2011.07942.x
6. Rabbit anti-IsdA: 10.1126/science.1081147
7. Rabbit anti-sortase A: 10.1126/science.1081147
8. Rabbit anti-SaeR: 10.1128/JB.00353-11
9. Mouse anti-His: by manufacturer, https://www.cellsciences.com/PDF/CSI20563.pdf
10. Mouse anti-LukAB (CC8-1-4.3.1.2.5.3, CC30-3-10.1.5.9, or CC45-1-11.3.5): by ELISA or immunoblot performed in this lab
11. Mouse anti-FLAG: by manufacturer and immunoblot performed in this lab
12. Rabbit anti-protein A: by manufacturer and immunoblot performed in this lab

Animals and other organisms

Policy information about studies involving animals; ARRIVE guidelines recommended for reporting animal research

Laboratory animals

Male C57BL/6J mice at 6-15 weeks of age were used in this study. All mice were fed and watered ad libitum with consistent access to food and water, and all mice were housed in a facility kept at ambient temperature and humidity with 12 hr light/12 hr dark cycles.

Wild animals

No wild animals were used in this study.

Field-collected samples

No field-collected samples were used in this study.

Ethics oversight

All experiments involving animals were reviewed and approved by the Institutional Animal Care and Use Committee of NYU Langone Health and were performed according to guidelines from the National Institutes of Health (NIH), the Animal Welfare Act, and US Federal Law.

Note that full information on the approval of the study protocol must also be provided in the manuscript.
